# Comprehensive list of preventative migraine headache medications without significant drug–drug interactions

**DOI:** 10.3389/fneur.2024.1527897

**Published:** 2024-12-12

**Authors:** Jay Dave, Ian Hakkinen, Pengfei Zhang

**Affiliations:** ^1^Department of Neurology, Mount Sinai Hospital, New York, NY, United States; ^2^Department of Neurology, Evergreen Health, Kirkland, WA, United States; ^3^Beth Israel Deaconess Medical Center, Harvard Medical School, Boston, MA, United States

**Keywords:** migraine, prevention medications, combinatorics, polypharmacy, drug–drug interaction

## Abstract

**Background/objective:**

Preventive medications are crucial in migraine prevention. In cases of refractory migraine headaches, multiple medications may be required. We seek to identify a comprehensive list of preventive migraine headache medications that can be used as two, three, and four drug combinations without drug–drug interactions.

**Methods:**

We compiled a list of prevention medications from Szperka et al.’s “Migraine Care in the Era of COVID-19” as well as American Headache Society’s 2018 and 2021 “Consensus Statements on Integrating New Migraine Treatments into Clinical Practice.” We obtained all possible two to four combinations of prevention medications through this list. We then filtered out all combinations containing at least one interaction based on DrugBank database and also identified least to most interacting medications.

**Results:**

A total of 26 unique prevention medications are identified. This results in a total of 325 combinations of two preventives, 2,600 combinations of three preventives, and 14,950 combinations of four preventives. There are a total of 124, 146, and 0 non-interacting two, three, and four preventive combinations, respectively. All except 16 combinations of pick-twos can be placed within a pick-three combinations. The resulting distinct non-interacting medications can be represented by a condensed list of 162 unique combinations of medications. CGRP antagonists, Botulinum toxin A, melatonin, and candesartan are least interacting.

**Conclusion:**

This list of migraine preventive medications without drug–drug interactions is a useful tool for clinicians seeking to manage refractory headaches more effectively by implementing an evidence-based polypharmacy.

## Introduction and literature review

Migraine is a highly prevalent and disabling disorder that affects about 12% of the population requiring acute and prevention therapy (1, 2). The American Headache Society (AHS) guidelines recommends prevention therapy for patients with frequent disabling migraine attacks; preventive medications reduce migraine frequency, severity, and may also improve quality of life (3, 4). Patients with primary headaches take an average of 4.37 medications; polypharmacy (five or more medications) occurs in 58.8% of patients with chronic migraine (5). Therefore, drug–drug interactions (DDI) must be considered when choosing multiple medications (6).

To our knowledge, there is no known study that exhaustively enumerates medication combinations for preventative treatment that are without potential for interactions. Following the methodology of Kaytser et al.’s study on non-interacting combinations of abortive migraine medications, the goal of our study is to use DrugBank, a comprehensive pharmaceutical database including both pharmacodynamic and pharmacokinetic interactions, to provide a rational tool for polypharmacy (7, 8). We further identified the least to most interacting medications.

## Methods

This project is composed of two phases: Phase 1 involves the identification and inclusion of prevention medication as well as the generation of all possible treatment combinations. Phase 2 involves the screening of these combined therapies into DrugBank for evaluations of interacting combinations.

### Phase 1

To identify a list of evidence-based prevention medications, we manually extracted prevention medications from American society guideline as well as Szperka et al.’s “Migraine Care in the Era of COVID-19: Clinical Pearls and Plea to Insurers.” This resulted in 26 medications (Table 1). We made the editorial decision to take out divalproex given that it is equivalent to valproic acid pharmacologically. Each medication was then converted to their respective RXID for DrugBank input purposes. Given this list of RXID, pick-two, pick-three, as well as pick-four combinations of RXID are algorithmically generated through custom codes. These combinations represent their respective combination therapies in DrugBank format. We then verified that the number of elements in each list corresponds to their respective value of combinatoric solutions.

**Table 1:** List of 26 unique migraine prevention medications and number of inclusion.

Drug name	Number of inclusions
Eptinezumab	40
Fremanezumab	40
Erenumab	40
Galcanezumab	40
OnabotulinumtoxinA	37
Melatonin	33
Candesartan	30
Cyproheptadine	28
Atenolol	24
Memantine	22
Lisinopril	16
Metoprolol	16
Nadolol	16
Valproate	16
Nebivolol	12
Carbamazepine	8
Propranolol	8
Timolol	8
Topiramate	8
Amitriptyline	4
Clonidine	4
Frovatriptan	4
Guanfacine	4
Pindolol	4
Venlafaxine	4
Zonisamide	4

### Phase 2

To identify DDI among pick two therapies, we input the pick-two list into DrugBank’s Application Programming Interface (API) to identify and generate a list of comprehensive pick-two pairings that contains at least one DDI (DrugBank’s API is essentially a portal allowing computers to access DrugBank data in bulk through an online interface). Notice that if any pick-three combinations involve a DDI, this implies that at minimum two of the three elements of that pick-three combination must contain a DDI. (For example, in the pick-three combinations of Valproate, Topiramate, and Zonisamide, we know that there exists at least one DDI given that the pick-two combination of Topiramate and Zonisamide constitutes a DDI.) With the comprehensive list of DDI for any given pick-two pairings, to obtain a list of pick-three combinations containing at minimum of one DDI, we simply compile a list of pick-three combinations which contains at least one pick-two DDI among any of its elements. The same logic can be applied to pick-four combination. We can then translate these results into their non-interacting counterparts by simply taking the difference between the original list of combinatoric results and the interacting list for pick- two, three, and four, respectively.

The results are manually verified by the first author (JD). During manual verification process, we identified that DrugBank’s record for candesartan is incomplete. The pick-two combinations as well as their DDI for candesartan were then manually obtained to compensate for the shortcoming.

Phase 1 and 2, both its textual manipulation as well as algorithmic access of DrugBank, were accomplished by custom written codes in both Python as well as Haskell.

### Hamiltonian path and drug selection

In clinical practice of refractory patients, when a selected pick two combination fails, it is maybe useful to be able to select the next combination of medications by changing only one mediation while still avoiding drug–drug interaction. To provide this sequential selection we performed a graph theory based Hamiltonian path analysis (see Table 2). A Hamiltonian path is a path that visits each node in the graph only once through these edges. We generate a mathematical graph using the following definitions, (1) Nodes of the graph are all pick two non-interacting drug combinations, (2) An edge connects two nodes that only have one drug difference. Through this Hamiltonian path, we were able to generate a sequential pick-two combination of medications that can be safely used without interactions.

**Table 2:** Hamiltonian pick 2 pathway.

atenolol	cyproheptadine
atenolol	Topiramate
fremanezumab	Topiramate
fremanezumab	Zonisamide
timolol	Fremanezumab
timolol	OnabotulinumtoxinA
timolol	melatonin
timolol	galcanezumab
timolol	erenumab
timolol	eptinezumab
eptinezumab	zonisamide
erenumab	zonisamide
galcanezumab	zonisamide
galcanezumab	venlafaxine
galcanezumab	valproate
valproate	nadolol
valproate	metoprolol
metoprolol	onabotulinumtoxinA
onabotulinumtoxinA	propranolol
nebivolol	onabotulinumtoxinA
nebivolol	melatonin
melatonin	nadolol
nadolol	onabotulinumtoxinA
memantine	onabotulinumtoxinA
melatonin	memantine
melatonin	metoprolol
lisinopril	melatonin
lisinopril	valproate
lisinopril	onabotulinumtoxinA
lisinopril	cyproheptadine
galcanezumab	lisinopril
galcanezumab	propranolol
galcanezumab	topiramate
galcanezumab	pindolol
galcanezumab	onabotulinumtoxinA
galcanezumab	nebivolol
galcanezumab	nadolol
galcanezumab	metoprolol
galcanezumab	memantine
galcanezumab	melatonin
galcanezumab	guanfacine
galcanezumab	frovatriptan
galcanezumab	cyproheptadine
galcanezumab	clonidine
galcanezumab	candesartan
galcanezumab	amitriptyline
fremanezumab	amitriptyline
fremanezumab	venlafaxine
fremanezumab	valproate
fremanezumab	propranolol
fremanezumab	pindolol
fremanezumab	onabotulinumtoxinA
fremanezumab	nebivolol
fremanezumab	nadolol
fremanezumab	metoprolol
fremanezumab	memantine
frovatriptan	memantine
fremanezumab	frovatriptan
fremanezumab	melatonin
fremanezumab	lisinopril
fremanezumab	guanfacine
fremanezumab	cyproheptadine
fremanezumab	clonidine
fremanezumab	candesartan
erenumab	candesartan
erenumab	venlafaxine
erenumab	valproate
erenumab	topiramate
erenumab	propranolol
erenumab	pindolol
erenumab	onabotulinumtoxinA
erenumab	nebivolol
erenumab	nadolol
erenumab	metoprolol
erenumab	memantine
erenumab	melatonin
erenumab	lisinopril
erenumab	guanfacine
erenumab	frovatriptan
erenumab	cyproheptadine
erenumab	clonidine
erenumab	amitriptyline
eptinezumab	amitriptyline
eptinezumab	venlafaxine
eptinezumab	valproate
eptinezumab	topiramate
eptinezumab	propranolol
eptinezumab	pindolol
eptinezumab	onabotulinumtoxinA
eptinezumab	nebivolol
eptinezumab	nadolol
eptinezumab	metoprolol
eptinezumab	memantine
eptinezumab	melatonin
eptinezumab	lisinopril
eptinezumab	guanfacine
eptinezumab	frovatriptan
eptinezumab	cyproheptadine
eptinezumab	clonidine
clonidine	memantine
candesartan	memantine
carbamazepine	candesartan
carbamazepine	galcanezumab
atenolol	carbamazepine
carbamazepine	fremanezumab
carbamazepine	erenumab
carbamazepine	eptinezumab
candesartan	eptinezumab
candesartan	venlafaxine
candesartan	topiramate
candesartan	onabotulinumtoxinA
atenolol	onabotulinumtoxinA
atenolol	valproate
atenolol	galcanezumab
atenolol	fremanezumab
atenolol	erenumab
atenolol	eptinezumab
atenolol	melatonin
candesartan	melatonin
candesartan	cyproheptadine
cyproheptadine	propranolol
cyproheptadine	nebivolol
cyproheptadine	nadolol
cyproheptadine	metoprolol

## Results

We identified a total of 26 unique prevention medications. Data access as well as conversion was finished on March 19, 2023. We find that there are a total of 325 combinations of pick two preventives, 2,600 combinations of pick three preventives, and 14,950 combinations of pick four preventives. After screening with DrugBank, we uncovered that there is a total of 124, 146, and 0 non-interacting pick two, pick three, and pick four preventive combinations, respectively. All non-interacting medications can be represented by a condensed list of 162 unique combinations of medications seen in Table 3. Out of these 124 combinations of pick-twos, all except 16 can be placed within a pick-three combinations. Table 1 lists the 26 unique migraine prevention medications and the number of non-interacting inclusions in our analysis. For example, topiramate is part of 8 non-interacting combinations as it only occurs 8 times in Table 1.

**Table 3:** Condensed list of unique combinations of medications.

amitriptyline	eptinezumab	
amitriptyline	erenumab	
amitriptyline	fremanezumab	
amitriptyline	galcanezumab	
atenolol	carbamazepine	eptinezumab
atenolol	carbamazepine	erenumab
atenolol	carbamazepine	fremanezumab
atenolol	carbamazepine	galcanezumab
atenolol	cyproheptadine	eptinezumab
atenolol	cyproheptadine	erenumab
atenolol	cyproheptadine	fremanezumab
atenolol	cyproheptadine	galcanezumab
atenolol	eptinezumab	melatonin
atenolol	eptinezumab	onabotulinumtoxinA
atenolol	eptinezumab	topiramate
atenolol	eptinezumab	valproate
atenolol	erenumab	melatonin
atenolol	erenumab	onabotulinumtoxinA
atenolol	erenumab	topiramate
atenolol	erenumab	valproate
atenolol	fremanezumab	melatonin
atenolol	fremanezumab	onabotulinumtoxinA
atenolol	fremanezumab	topiramate
atenolol	fremanezumab	valproate
atenolol	galcanezumab	melatonin
atenolol	galcanezumab	onabotulinumtoxinA
atenolol	galcanezumab	topiramate
atenolol	galcanezumab	valproate
candesartan	carbamazepine	eptinezumab
candesartan	carbamazepine	erenumab
candesartan	carbamazepine	fremanezumab
candesartan	carbamazepine	galcanezumab
candesartan	cyproheptadine	eptinezumab
candesartan	cyproheptadine	erenumab
candesartan	cyproheptadine	fremanezumab
candesartan	cyproheptadine	galcanezumab
candesartan	eptinezumab	melatonin
candesartan	eptinezumab	memantine
candesartan	eptinezumab	onabotulinumtoxinA
candesartan	eptinezumab	topiramate
candesartan	eptinezumab	venlafaxine
candesartan	erenumab	melatonin
candesartan	erenumab	memantine
candesartan	erenumab	onabotulinumtoxinA
candesartan	erenumab	topiramate
candesartan	erenumab	venlafaxine
candesartan	fremanezumab	melatonin
candesartan	fremanezumab	memantine
candesartan	fremanezumab	onabotulinumtoxinA
candesartan	fremanezumab	topiramate
candesartan	fremanezumab	venlafaxine
candesartan	galcanezumab	melatonin
candesartan	galcanezumab	memantine
candesartan	galcanezumab	onabotulinumtoxinA
candesartan	galcanezumab	topiramate
candesartan	galcanezumab	venlafaxine
candesartan	melatonin	memantine
candesartan	memantine	onabotulinumtoxinA
clonidine	eptinezumab	memantine
clonidine	erenumab	memantine
clonidine	fremanezumab	memantine
clonidine	galcanezumab	memantine
cyproheptadine	eptinezumab	lisinopril
cyproheptadine	eptinezumab	metoprolol
cyproheptadine	eptinezumab	nadolol
cyproheptadine	eptinezumab	nebivolol
cyproheptadine	eptinezumab	propranolol
cyproheptadine	erenumab	lisinopril
cyproheptadine	erenumab	metoprolol
cyproheptadine	erenumab	nadolol
cyproheptadine	erenumab	nebivolol
cyproheptadine	erenumab	propranolol
cyproheptadine	fremanezumab	lisinopril
cyproheptadine	fremanezumab	metoprolol
cyproheptadine	fremanezumab	nadolol
cyproheptadine	fremanezumab	nebivolol
cyproheptadine	fremanezumab	propranolol
cyproheptadine	galcanezumab	lisinopril
cyproheptadine	galcanezumab	metoprolol
cyproheptadine	galcanezumab	nadolol
cyproheptadine	galcanezumab	nebivolol
cyproheptadine	galcanezumab	propranolol
eptinezumab	frovatriptan	memantine
eptinezumab	guanfacine	
eptinezumab	lisinopril	melatonin
eptinezumab	lisinopril	onabotulinumtoxinA
eptinezumab	lisinopril	valproate
eptinezumab	melatonin	memantine
eptinezumab	melatonin	metoprolol
eptinezumab	melatonin	nadolol
eptinezumab	melatonin	nebivolol
eptinezumab	melatonin	timolol
eptinezumab	memantine	onabotulinumtoxinA
eptinezumab	metoprolol	onabotulinumtoxinA
eptinezumab	metoprolol	valproate
eptinezumab	nadolol	onabotulinumtoxinA
eptinezumab	nadolol	valproate
eptinezumab	nebivolol	onabotulinumtoxinA
eptinezumab	onabotulinumtoxinA	propranolol
eptinezumab	onabotulinumtoxinA	timolol
eptinezumab	pindolol	
eptinezumab	zonisamide	
erenumab	frovatriptan	memantine
erenumab	guanfacine	
erenumab	lisinopril	melatonin
erenumab	lisinopril	onabotulinumtoxinA
erenumab	lisinopril	valproate
erenumab	melatonin	memantine
erenumab	melatonin	metoprolol
erenumab	melatonin	nadolol
erenumab	melatonin	nebivolol
erenumab	melatonin	timolol
erenumab	memantine	onabotulinumtoxinA
erenumab	metoprolol	onabotulinumtoxinA
erenumab	metoprolol	valproate
erenumab	nadolol	onabotulinumtoxinA
erenumab	nadolol	valproate
erenumab	nebivolol	onabotulinumtoxinA
erenumab	onabotulinumtoxinA	propranolol
erenumab	onabotulinumtoxinA	timolol
erenumab	pindolol	
erenumab	zonisamide	
fremanezumab	frovatriptan	memantine
fremanezumab	guanfacine	
fremanezumab	lisinopril	melatonin
fremanezumab	lisinopril	onabotulinumtoxinA
fremanezumab	lisinopril	valproate
fremanezumab	melatonin	memantine
fremanezumab	melatonin	metoprolol
fremanezumab	melatonin	nadolol
fremanezumab	melatonin	nebivolol
fremanezumab	melatonin	timolol
fremanezumab	memantine	onabotulinumtoxinA
fremanezumab	metoprolol	onabotulinumtoxinA
fremanezumab	metoprolol	valproate
fremanezumab	nadolol	onabotulinumtoxinA
fremanezumab	nadolol	valproate
fremanezumab	nebivolol	onabotulinumtoxinA
fremanezumab	onabotulinumtoxinA	propranolol
fremanezumab	onabotulinumtoxinA	timolol
fremanezumab	pindolol	
fremanezumab	zonisamide	
frovatriptan	galcanezumab	memantine
galcanezumab	guanfacine	
galcanezumab	lisinopril	melatonin
galcanezumab	lisinopril	onabotulinumtoxinA
galcanezumab	lisinopril	valproate
galcanezumab	melatonin	memantine
galcanezumab	melatonin	metoprolol
galcanezumab	melatonin	nadolol
galcanezumab	melatonin	nebivolol
galcanezumab	melatonin	timolol
galcanezumab	memantine	onabotulinumtoxinA
galcanezumab	metoprolol	onabotulinumtoxinA
galcanezumab	metoprolol	valproate
galcanezumab	nadolol	onabotulinumtoxinA
galcanezumab	nadolol	valproate
galcanezumab	nebivolol	onabotulinumtoxinA
galcanezumab	onabotulinumtoxinA	propranolol
galcanezumab	onabotulinumtoxinA	timolol
galcanezumab	pindolol	
galcanezumab	zonisamide	

Our analysis revealed that the top medications with the most inclusion in the non-interacting list are: (1) CGRP inhibitors class, (2) OnabotulinumtoxinA, (3) Melatonin, and (4) Candesartan. The least number of inclusions in the non-interacting list were tied among Amitriptyline, Clonidine, Guanfacine, Pindolol, Venlafaxine, and Zonisamide with four non-interacting inclusions each. From our examination of non-interacting combinations in Table 3, it became evident that drugs belonging to the same category, such as beta-blockers and antidepressants, do not appear together in any of these combinations. Additionally, medications with distinct mechanisms of action but similar effects, such as a broader range of antihypertensive drugs, are absent from Table 3. This observation underscores that our analysis does not rely solely on combinatorial mathematics but also factors in the theoretical drug interactions between these medications.

We provide multiple choices for two non-interacting drug combinations in Table 2; if the selected combination fails, the next combination of medications can be chosen while still avoiding drug–drug interaction. Table 2 lists the two drug combinations in a sequential manner with the following characteristics: (1) Consecutive entries differing only by one medication. (2) One can start anywhere in Table 2 and move to the following consecutive choice of medication when the former selection fails.

## Discussion

We present a list of 26 unique headache preventatives and their combinations without any drug–drug interactions based on the DrugBank database (9). Our intention is to provide a tool to help guide decision making in choosing effective, non-interacting, and rational combination therapy. Although we proposed a list of unique combinations without drug–drug interactions, our intention is not to encourage polypharmacy, but to explore the safety of combination therapies. Our discussion below will follow specific classes of prevention medications.

### Calcitonin gene-related peptide (CGRP)-targeted monoclonal antibodies

CGRP is a neuropeptide widely distributed throughout the body, serving various functions, including sensory, vascular, and immune modulation (10). In the trigeminal system, CGRP is highly abundant in small sensory fibers and interacts with receptors like the CGRP receptor and AMY1 receptor, leading to vasodilation and neurogenic inflammation (11). Blockade of CGRP through small molecules or monoclonal antibodies represents an effective treatment option for migraine prevention (3).

This class of medications has the most representation in our list. Galcanezumab, Erenumab, Fremanezumab, and Eptinezumab were each included 40 times in the non-interacting combinations. Several factors might account for the high inclusion list: The CGRP antibodies are metabolized by degradation into peptides and single amino acids, which could account for their low risk of drug–drug interactions (12, 13). The mechanism of action likely occurs outside the blood–brain barrier, involving regions like neural ganglia, dura, and various brain regions (14). This class of medication has been specifically designed for migraine and exert a more direct effect on migraine specific pathways compared to other prevention drugs (15). They are also preferred as a first-line treatment for patients who want to avoid oral prophylactics owing to potential adverse effects, drug–drug interactions, or slower onset of action (16).

### Onabotulinumtoxin A

Onabotulinumtoxin A is a complex involving a 150-kDa botulinum neurotoxin and neurotoxin-associated proteins (NAPs) (17). It operates at peripheral nerve terminals, disrupting synaptic vesicle cycles by cleaving SNAP-25, a key SNARE protein (18, 19). This inhibits vesicle fusion and neurotransmitter release, affecting proteins and receptors like TRPV1, TRPA1, P2X3, substance P, glutamate, and CGRP (20–22). Cleaved SNAP-25 forms nonfunctional SNARE complexes within neurons due to specific interactions, contributing to the toxin’s long-lasting effects (about 3–4 months in motor nerves, 6–9 months in autonomic nerves) (23–25). Ubiquitination of the toxin’s light chain restores neurotransmission, and the presence of sprouts in motor neurons during recovery may influence the duration in specific nerve and tissue targets (26–28). Multiple pericranial injections are needed to target extracranial and intracranial nerves, such as trigeminal and cervical nerves, respectively, and reduce the hyperexcitability of these neurons involved in the migraine pathway (17, 29, 30).

Onabotulinumtoxin A is the second most included medication class in our non-interacting combination list, with 37 inclusions. Given its topical nature, we might be overstating its interactions in DrugBank. For example, Amitriptyline and Botox are theoretically contraindication due to dual blockade of “Botulinum Toxin Type A’s ability to inhibit acetylcholine release [which] may produce additive effects when used concomitantly with anticholinergic agents (31).” However, this is not a clinically relevant interaction as the latter is topical. Nevertheless, onabotulinumtoxin A has a relatively mild treatment related adverse event profile, the PREEMPT and COMPEL studies provide some reassurance of the safety and tolerability of Botox in clinical practice with concomitant oral prophylaxis (32–35).

### Melatonin

Melatonin offers potential benefits for migraine management by regulating neurotransmitters and neural pathways, suppressing CGRP release to control brain blood flow, and acting as an analgesic by increasing *β*-endorphin release, activating melatonin receptors, and potentially inhibiting pain-producing substances. Its anxiolytic and antidepressant properties further help alleviate migraine-related pain through its influence on various pathways (36). Melatonin is metabolized primarily by hepatic CYP1A2, so most drug–drug interactions occur when other agents are metabolized by same enzyme (37). Melatonin interacts with opioid analgesics by potentiating their effect; therefore, it should be used with caution in patients taking and/or overusing opioids (38). Its specific site of action and CYP enzyme needed for clearance is likely one of the reasons why it has more inclusions in our list.

### Candesartan

Its mechanism as a migraine prophylactic is thought to work by reducing the effects of angiotensin II, which can have various effects relevant to migraine, including vasoconstriction, increased sympathetic activity, and catecholamine release (39). Angiotensin II, in addition to its systemic role, is involved in local functions, including within the brain. It acts through the AT1 receptor to modulate cerebrovascular flow, impact fluid and electrolyte balance, influence autonomic pathways, and affect neuroendocrine systems (40). Angiotensin II is also thought to modulate potassium channels and calcium activity in cells, and can impact neurotransmitters like dopamine, and serotonin metabolites (39, 41, 42). Additionally, it activates nuclear factor kappa B, potentially influencing nitric oxide synthase expression (43, 44). Candesartan is primarily eliminated unchanged in the urine, a minor portion of candesartan (less than 20%) undergoes hepatic metabolism through cytochrome P450 2C9, resulting in an inactive metabolite (45). Candesartan is the 4th least interacting medication. Although we are unclear as to why it is least interacting, we hypothesize that given its anti-hypertensive property as the chief one leading to DDI, the process of elimination can be a potential reason why it has less drug–drug interactions as compared to the agents cleared hepatically.

### Cyproheptadine

Cyproheptadine mechanism of action is by directly inhibiting the release of histamine and serotonin, by competitively and reversibly blocking their actions at receptor sites (46). This unique mechanism leads to inhibiting the release of vasoactive peptides, including calcitonin gene-related peptides, while simultaneously preventing the activation of serotonin 1B and 1D receptors and the development of neurogenic inflammation triggered by trigeminal nerve stimulation (47). Cyproheptadine’s metabolism leads to the formation of a unique quaternary ammonium glucuronide conjugate metabolite in human urine (48). This distinctive metabolite minimizes potential interactions with commonly used migraine medications that share similar pathways. However, medications with similar mechanisms of action can lead to DDI. For example, cyproheptadine’s anticholinergic and antiserotonergic properties (46) can augment the anticholinergic effects of amitriptyline, leading to increased side effects such as dry mouth, blurred vision, and constipation.

### Atenolol

Beta-blockers are believed to primarily exert their effects centrally. Their main mechanisms of action involve blocking β1-mediated effects, leading to the inhibition of sodium release and tyrosine hydroxylase activity (49). Beta-blockers also reduce the firing rate of noradrenergic neurons in the locus coeruleus, regulate the firing rate of neurons in the periaqueductal gray (PAG), and potentially interact with the serotonergic system by blocking 5-HT2C and 5-HT2B receptors (50). Some hypothesize that beta-blockers may achieve their prophylactic effects in migraine by acting on the ventroposteromedial thalamic nucleus and inhibiting cortical spreading depression (50). Atenolol has more inclusions in our study compared to other beta-blockers such as nebivolol or propranolol due to its metabolic clearance. It is mainly eliminated by the kidneys and about 5% is cleared by the liver (51). Whereas nebivolol and propranolol which are metabolized mainly by the cytochrome P450 enzyme, strong inducers such as carbamazepine can decrease their efficacy (52, 53).

### Most common interacting drugs

Most common mechanism of kinetic drug–drug interactions are due to either induction or inhibition of the cytochrome P450 (CYP) system (5). It has been shown that the main enzyme, CYP2D6 cytochrome P450, is responsible for converting venlafaxine to desvenlafaxine (active form). It is also responsible for the conversion of other antidepressants such as amitriptyline and topiramate. This shared metabolism with the other drugs can lead to a reduced net drug effect of venlafaxine due to decreased conversion to its active form. This may be the reason that topiramate, when added to venlafaxine for migraine management, can sometimes seem to make depression worse (54). Additionally, it has been shown that beta-blockers can also interact by decreasing conversion of venlafaxine to desvenlafaxine (54). Amitriptyline and MAOIs may exacerbate psychosis symptoms in patients with unipolar disorder with psychotic features (55).

Guanfacine is sensitive to drug–drug interactions perpetrated by strong inhibitors and inducers of CYP3A4 (56). Drugs like amitriptyline which is alpha-1 receptor antagonist, can oppose the action of adrenergic agonist such as guanfacine. Therefore, coadministration of these two drugs can cancel out each other’s effect (57).

We observe that pindolol is the least non-interacting beta-blocker. This might be because pindolol has a non-selective affinity for beta-1 and beta-2 receptors, which is stimulated by catecholamines having effect on various sites in the body, such as the central nervous system, respiratory system, and the heart (58). Furthermore, pindolol exhibits beta-adrenergic partial agonism and 5-HT1A receptor antagonism properties. The combination of pindolol with SSRIs results in a shorter response time to treatment compared with SSRIs alone (59). This drug association improvement is attributed to a decrease in the 5-HT1A-autoreceptor-mediated inhibitory feedback of serotonergic pathways by pindolol (60).

Frovatriptan is primarily metabolized by CYP1A2, so use of its inhibitors such as carbamazepine should be avoided (61). Triptans are also serotonin agonists that work on the 5HT1B and 5HT1D receptors and concomitant use with SSRI/SNRI has a small chance of leading to serotonin syndrome which is unsubstantiated in clinical practice (62).

Clonidine, an alpha-2 agonist, can interact with several antidepressants such as TCAs’, which can lead to blockade of norepinephrine reuptake (62).

Zonisamide is very similar to that of topiramate. Both drugs seem to work through various mechanisms, including blocking voltage sodium channels, inhibiting carbonic anhydrase enzyme, enhancing GABA release, modulating serotoninergic and dopaminergic neurotransmission, and inhibiting potassium-mediated release of glutamate (63). Notably, zonisamide, unlike topiramate, also blocks T-type calcium channels (64). Given how broad acting both their mechanisms are, it likely explains the low inclusion count in our study, and zonisamide’s activity on calcium channels leads to interaction with medications such as candesartan, further increasing DDI count compared to topiramate.

### Hamiltonian cycle

In our approach to migraine prevention, we have employed Hamiltonian cycle analysis from graph theory, a systematic method that sequentially selects non-interacting medication pairs (Table 2). This approach offers several advantages, primarily in reducing the risk of adverse drug interactions, which is particularly crucial when managing migraine patients on multiple medications. The method’s systematic and algorithmic nature ensures exhaustive exploration of medication combinations, potentially optimizing treatment plans for individual patients. Additionally, its adaptability allows for the selection of alternative medication pairs if the initial choice proves ineffective, enabling personalized treatment adjustments. However, it is vital to acknowledge its limitations, such as oversimplification of complex drug interactions and individual patient responses. Migraine treatment involves multifaceted factors, including patient-specific physiology, genetics, and drug mechanisms, which may not be fully captured by Hamiltonian cycles. Furthermore, a focus solely on non-interaction may inadvertently exclude potentially beneficial drug pairs with mild interactions, necessitating a careful balance between safety and efficacy considerations. Lastly, the accuracy of outcomes is contingent on the quality of data used to construct the interaction graph and the appropriateness of the chosen model for Hamiltonian cycle analysis, making thorough data validation essential for optimal treatment recommendations in migraine prophylaxis.

### Limitations

Much like the Kaytser study (7), our investigation is limited by our selection of included medications. We curated a roster of preventive medications, drawing from Szperka et al.’s “Migraine Care in the Era of COVID-19” and incorporating the 2018 and 2021 Consensus Statements of the American Headache Society; we acknowledge that our colleagues may have favored medications that we omitted. However, the compilation of an exhaustive list falls beyond the scope of our project. Our principal objective is to delineate a methodological approach and offer a practical compendium of valuable medication combinations.

It is important to note that DrugBank is conservative with regards to drug–drug interactions. For example, based on DrugBank, Frovatriptan is known to produce hypertension, so if used in combination with antihypertensive agents used in this study, there could be a decrease in the antihypertensive effects of the antihypertensive agents (65). This is listed as a minor interaction but is a reason why Frovatriptan inclusion number was four. Our approach to querying DrugBank lacks the capacity to appraise the severity of drug interactions, prompting the necessity for an organized classification system. Regrettably, our current methodology lacks the requisite computational tools for this purpose, thereby confining the scope of our study.

Recent advances in pharmacogenetics and pharmacogenomics studies have made an impact on our understandings of drug–drug interaction. For example, Lionetto et al. has suggested that “[pharmacogenomics] has provided a novel tool to understand the basics of the DDIs. The DDIs, based on the sharing of same pathways among different drugs, can be elided or exacerbated by the individual genetic make-up, so that characterization of the genotype might be crucial to make appropriate choice of combination therapies” (66). Our study on DDI in the DrugBank database does not take into account this level of personalization. (Although it should be noted that DrugBank is one of the databases which contains pharmacogenomic data (66).) Far from excluding these novel advances, it is our hope that with increase in computational power and advance in personalized medicine, that our approach may be applicable for individual patients: using pharmacogenetics and genomics, specific DDI maybe marked as present/absent based on genetic profile, thereby allowing for the list in Table 3 to be expanded/reduced for different patients.

Along the same line, comorbidities and concurrently used medications for those comorbidities are not taken into account in this study. In a patient with type 2 diabetes and renal failures, for example, the list of possible medications as well as their DDIs will be dramatically less than what is proposed in Table 3. Practical uses of Table 3 therefore must require clinicians to screen out contra-indicated medication combinations for the individual patient. Furthermore, we encourage clinicians to not use Table 3 blindly but to consider the clinical context of each patient’s existing medication list. Finally, we believe that the most optimum way to avoid DDI in clinical practice is the principle of pursuing/finding the optimum monotherapy first (67).

Furthermore, it’s worth noting that our study may exhibit bias toward newer medications, given the potential for unforeseen drug–drug interactions (DDI) with established drugs. For instance, the long-term implications of CGRP blockade remain uninvestigated, warranting a thorough investigation of cardio- and cerebrovascular safety, considering the proposed involvement of CGRP in human coronary arteries (68).

Lastly, we did not assess the influence of the duration of drug combination usage on adverse outcomes. Nevertheless, we posit that heightened frequency of use may escalate the risk of interactions, necessitating further exploration.

### Future directions

To gain a more comprehensive understanding of potential drug–drug interactions in the realm of preventive migraine medications, a thorough study compiling a list of side effects associated with specific drug combinations is essential. This initiative could shed light on the tolerability of these interactions and their clinical significance.

In situations where patients require combinations of four or more drugs for effective migraine prevention, there is a need for further research to identify which combinations offer the most favorable profiles in terms of side effects. Such investigations can play a crucial role in refining treatment strategies for patients managing complex medication regimens.

Considering the multifaceted nature of migraine management, future research can delve into exploring interactions between acute and preventive medications. This approach is poised to enrich our understanding of how these aspects interact, offering insights into optimizing comprehensive migraine treatment strategies.

Additionally, the pursuit of longitudinal studies represents a promising avenue to evaluate the real-world safety and efficacy of preventive migraine medications. These studies would involve continuous, long-term monitoring and assessment of patients, providing a deeper understanding of the sustained benefits and potential risks associated with these medications. In turn, this research has the potential to offer valuable insights into the long-term management of migraines and guide informed treatment decisions.

Finally, even though direct clinical and empirical validation of our combinatoric approach may be challenging when done as a single study—the number of subjects needed to evaluate for validity of individual DDI among 325, 2,600, or 14,950 combinations would quickly become astronomical—critical appraisal of DrugBank’s description of individual pairings of interactions for migraine would be a vital and important future undertaking as a literature or meta-analysis project. Such an endeavor may allow us to describe specific interactions in DrugBank as probability of occurrences, which in turn, may allow us to generate a probabilistic version Table 3.

## Conclusion

This list of migraine preventive medications without drug–drug interactions is a useful tool for clinicians seeking to manage refractory headaches more effectively by implementing an evidence-based polypharmacy.

## Data Availability

The raw data supporting the conclusions of this article will be made available by the authors, without undue reservation.
